# Microsatellite based genetic diversity study in indigenous chicken ecotypes of Karnataka

**DOI:** 10.14202/vetworld.2015.970-976

**Published:** 2015-08-12

**Authors:** B. H. Rudresh, H. N. N. Murthy, M. R. Jayashankar, C. S. Nagaraj, A. M. Kotresh, S. M. Byregowda

**Affiliations:** 1 Department of Animal Genetics and Breeding, Veterinary College, Karnataka Veterinary, Animal and Fisheries Sciences University, Shimoga, Karnataka, India; 2 Department of Livestock Production and Management, Veterinary College, Karnataka Veterinary, Animal and Fisheries Sciences University, Bangalore, Karnataka, India; 3 Department of Animal Genetics and Breeding, Veterinary College, Karnataka Veterinary, Animal and Fisheries sciences University, Bangalore, Karnataka, India; 4 All India Coordinated Research Project on Poultry Meat, Karnataka Veterinary, Animal and Fisheries Sciences University, Veterinary College, Bangalore, Karnataka, India; 5 Department of Veterinary Physiology and Biochemistry, Veterinary College, Karnataka Veterinary, Animal and Fisheries sciences University, Shimoga, Karnataka, India; 6 Institute of Animal Health & Veterinary Biologicals, Bangalore, Karnataka Veterinary, Animal and Fisheries Sciences University, Karnataka, India

**Keywords:** animal genetic resources, ecotypes, microsatellites, polyacrylamide gel

## Abstract

**Aim:**

The current study was the first of its kind taken upon indigenous ecotypes of the Karnataka in order to unravel the diversity details at 20 chicken microsatellite regions.

**Materials and Methods:**

210 indigenous chicken belonging to six districts of Bangalore and Mysore division formed the target sample for the present study. The genomic deoxyribonucleic acid was isolated by phenol chloroform isoamyl alcohol method. A panel of 20 microsatellite regions, including 14 recommended by FAO and six identified from published scientific literature became the targeted chicken genomic region. 27-33 samples were successfully genotyped in each of the six ecotypes through simplex or multiplex polymerase chain reactions, polyacrylamide gel electrophoresis and silver staining for the selected microsatellite panel.

**Results:**

The chickens of Ramanagara and Chamrajnagara were most distant with a Nei’s genetic distance value of 0.22. The chickens of Bangalore rural and Mysore were least distant with a value of 0.056. The Ramanagara and Chamrajnagara pair had Nei’s genetic identity value of 0.802, which is least among all pairs of ecotypes. There were five main nodes from which the six ecotypes evolved on the basis 20 microsatellite markers used in this study. This study indicates that the four ecotypes Ramnagara, Bangalore Rural, Chickaballapura and Mysore are genetically identical due to their common ancestral evolution while, Mandya and Chamrajnagara ecotypes formed a relatively different cluster due to a separate common ancestral chicken population and less number of generations since drifting from bifurcation node.

**Conclusion:**

Twenty microsatellite markers based genetic diversity study on six indigenous ecotypes indicated lower genetic distances as well as lower F_ST_ values compared to the distinguished breeds reported. There were two main clusters, which differentiated into six ecotypes. They may differentiate into more distinct varieties if bred in isolation for a longer number of generations.

## Introduction

Characterization of animal genetic resources (AnGR) involves activities associated with the identification, quantitative and qualitative description, documentation of breed populations, the natural habitats and production systems to which they are not adapted. Since the beginning of the 1990s, molecular data have become more and more relevant for the characterization of genetic diversity [1]. In 1993, FAO working group proposed a global program for characterization of AnGR, including molecular genetic characterization and formulated the secondary guidelines: Measurement of domestic animal diversity.

Indigenous chicken have an inherent scavenging and nesting habit. Years of natural selection under scavenging conditions, has made them tough and resistant to various diseases, especially to those caused by bacteria, and protozoa and other internal and external parasites; they have better survival than the commercial hybrid strains under village production conditions. However, the village chicken is a poor egg producer, laying on average 40-60 eggs per year in three or four clutches, with an average egg weight of around 35-45 g. They generally have small body size; for various chicken breeds, mature body weight varies between 1.3 and 1.9 kg for males and between 1.0 and 1.4 kg for females.

Molecular genetic characterization explores polymorphism in selected protein molecules and deoxyribonucleic acid (DNA) markers in order to measure genetic variation at the population level. To obtain a better knowledge of AnGR, of their present and potential future uses for food and agriculture in defined environments, and their current state as distinct breed populations. In poultry, different genetic marker systems have successfully been used for estimation of genetic variability. They were DNA fingerprints, RAPDs, and microsatellites. Recently, the determination of heterozygosity and genetic distances based on microsatellite markers is regarded as a most convenient tool, and many microsatellite loci are available in chicken. These tandem repeated DNA segments show extensive allelic differences in length based mainly on variation in the number of repeats and, partly, in adjacent regions. These studies are important for understanding the history of species, and for testing hypotheses regarding evolutionary processes. Currently, microsatellites are most commonly used, and successful application of this sort of markers in diversity studies has been reported for all major livestock species [2-6]. Microsatellites have proven to be an extremely valuable tool for genome mapping in many organisms, but their applications span over different areas ranging from forensic DNA studies to population genetics and conservation/management of biological resources.

The Indian chicken ecotypes consisted of different phenotypes of almost centuries of natural selection and reared by smallholder farmers across distinct agro-ecological regions. To date, however, no investigation has yet been directed toward the use of microsatellite markers to study the genetic variability of these ecotypes, despite a large number of microsatellite primers published in scientific literature. Despite the importance of native chicken in tribal/rural areas, information is lacking on their genetic makeup with respect to genetic variability, genetic relationships, performance, adaptability and resistance to diseases. In this context, this study of diversity in indigenous chicken ecotypes based on appropriate microsatellite panel in Karnataka was taken up.

## Materials and Methods

### Ethical approval

This research was approved by the Local Ethics Committee of Veterinary College, Bangalore, Karnataka Veterinary, Animal and Fisheries sciences University, as per LPM/IAEC/77/2011 dated 20-01-2011.

### Experimental birds

**Figure-1:**
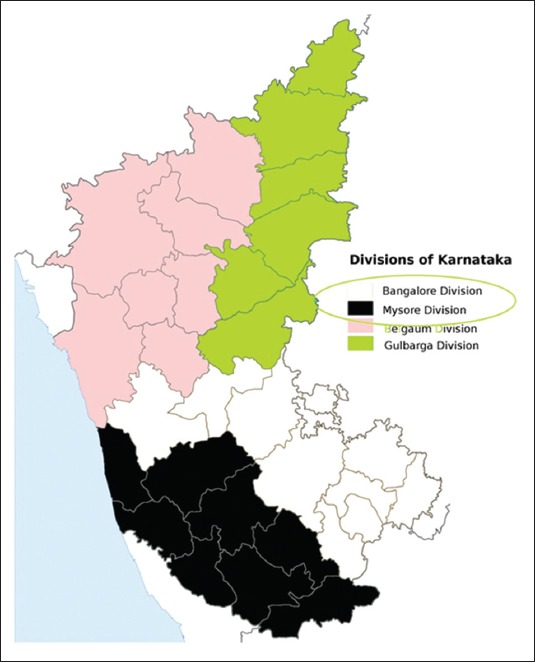
Study area map

The indigenous chicken belonging to Ramnagara, Bangalore rural and Chikkaballapur districts of Bangalore division and Chamarajnagara, Mysore and Mandya districts of Mysore division of Karnataka state (Figure-1) maintained at AICRP on Poultry for Meat, Veterinary College, Hebbal formed the material for the present study. 35 randomly chosen adult birds from each of the above districts formed the research sample for the present study.

The birds belonging to the six districts were wing banded at the hatch from randomly collected fertile eggs in respective districts. The birds chosen in each district belonged to three hatches and were badged while allocating to breeding pens after growing phase. The wing bands and serial numbers were noted down at the time of blood collection from each bird. The serial numbers for each district were maintained on the 2 ml Eppendorf tubes and subsequent tubes until the successful amplification of desired segment in each marker. The numbers were maintained until genotyping and further statistical analysis.

### Primer and DNA collection

20 primer pairs corresponding to microsatellite regions of chicken autosomes 1, 2, 3, 4, 5, 6, 7, 8, 10, 13, 14, 17, and 23 were identified by review of literature (Table-1). The procedure recommended by Khosravinia *et al*. [2] with little modifications for DNA extraction from whole fresh avian blood was adopted.

**Table-1:** The microsatellite primer details.

Marker name	F/R	Primer sequence (5’-3’)	Primer length	Chromosome number	At (°C)	Product size (bp)
ADL0020	F R	GCACTCAAAAGAAAACAAAT TAGATAAAAATCCTTCCCTT	20 20	1	55	88
ADL0023	F R	CTTCTATCCTGGGCTTCTGA CCTGGCTGTGTATGTGTTGC	20 20	5	60	164
ADL0176	F R	TTGTGGATTCTGGTGGTAGC TTCTCCCGTAACACTCGTCA	20 20	2	52	175-194
MCW007	F R	AGCAAAGAAGTGTTCTCTGTTCAT ACCCTGCAAACTGGAAGGGTCTCA	24 24	1	60	313-349
MCW0 041	F R	CCCATGTGCTTGAATAACTTGGG CCAGATTCTCAATAACAATGGCAG	23 24	2	55	140-162
MCW0014	F R	TATTGGCTCTAGGAACTGTC GAAATGAAGGTAAGACTAGC	20 20	6	58	164-182
MCW0183	F R	ATCCCAGTGTCGAGTATCCGA TGAGATTTACTGGAGCCTGCC	21 21	7	58	296-326
ADL0278	F R	CCAGCAGTCTACCTTCCTAT TGTCATCCAAGAACAGTGTG	20 20	8	60	114-126
MCW0067	F R	GCACTACTGTGTGCTGCAGTTT GAGATGTAGTTGCCACATTCCGAC	22 24	10	60	176-186
MCW0104	F R	TAGCACAACTCAAGCTGTGAG AGACTTGCACAGCTGTGTACC	21 21	13	60	190-234
MCW0123	F R	CCACTAGAAAAGAACATCCTC GGCTGATGTAAGAAGGGATGA	21 21	14	60	76-100
MCW0330	F R	TGGACCTCATCAGTCTGACAG AATGTTCTCATAGAGTTCCTGC	21 22	17	60	256-300
MCW0165	F R	CAGACATGCATGCCCAGATGA GATCCAGTCCTGCAGGCTGC	21 20	23	60	114-118
MCW0103	F R	AACTGCGTTGAGAGTGAATGC TTTCCTAACTGGATGCTTCTG	21 21	3	64	266-270
MCW0034	F R	TGCACGCACTTACATACTTAGAGA TGTCCTTCCAATTACATTCATGGG	24 24	2	60	212-246
MCW0081	F R	GTTGCTGAGAGCCTGGTGCAG CCTGTATGTGGAATTACTTCTC	21 22	5	60	112-135
MCW0284	F R	GCCTTAGGAAAAACTCCTAAGG CAGAGCTGGATTGGTGTCAAG	22 21	4	60	235-243
MCW0078	F R	CCACACGGAGAGGAGAAGGTCT TAGCATATGAGTGTACTGAGCTTC	22 24	5	60	135-147
ADL0268	F R	CTCCACCCCTCTCAGAACTA CAACTTCCCATCTACCTACT	20 20	1	60	102-116
ADL0112	F R	GGCTTAAGCTGACCCATTAT ATCTCAAATGTAATGCGTGC	20 20	10	58	120-134

### Polymerase chain reaction (PCR) details

PCR reactions were set up on clean benches with autoclaved lab disposables like micro tips, PCR tubes, 1.5-2.0 ml Eppendorf tubes and autoclaved milliq water ensuring proper thawing, mixing of PCR reaction components and cold chain of around 4°C. The standard PCR protocol was followed for amplifying all the microsatellite regions. The standard 12% non-denaturing polyacrylamide gel (PAGE) was performed for detection of allele size on all PCR products belonging to 20 microsatellite loci.

### Genotyping

The silver staining was adopted for staining of PAGEs in this study. The improved staining method developed by Halima *et al*. [7]. The same technique with little modification was adopted in this study. Each of the lanes with clearer bands was compared with the 50 bp ladder lane to genotype each sample.

### Statistical analysis

The data obtained regarding genotypes of 20 microsatellite regions for six ecotypes of Bangalore and Mysore divisions in base pair format was utilized as input file for GenAlex 6.5b5 in order to obtain genetic distances, and Wright’s F-statistics. The dendrogram was obtained from the POPGENE software. The Polymorhic information for each marker was obtained from MS tools programme.

## Results and Discussion

### Polymorphic information content (PIC) values

The PIC values of 20 loci for all the six ecotypes are presented in the Table-2. The locus MCW0183 was the least polymorphic (0.219-0.5846) while MCW0103 was the most polymorphic (0.8324-0.894) locus across six ecotypes.

**Table-2:** Microsatellite loci wise PIC values for six ecotypes.

Locus	Ecotypes					

	RNGR	BNGRRL	CBR	MYSORE	MANDYA	CHMNGR
ADL020	0.7521	0.6220	0.7362	0.6445	0.6513	0.7494
ADL023	0.7622	0.6588	0.7216	0.7024	0.7045	0.7846
ADL176	0.6487	0.4463	0.6392	0.5812	0.5945	0.6774
MCW007	0.8523	0.7938	0.8727	0.8053	0.8272	0.8386
MCW041	0.7017	0.6530	0.7440	0.7577	0.7366	0.7923
MCW014	0.8373	0.7739	0.8367	0.7829	0.7568	0.7990
MCW183	0.4034	0.2190	0.4845	0.3859	0.3907	0.5846
ADL278	0.7814	0.6302	0.6933	0.7225	0.7801	0.8115
MCW067	0.7252	0.6018	0.7067	0.6998	0.6423	0.6838
MCW104	0.7592	0.6937	0.7984	0.6476	0.7307	0.8350
MCW123	0.6041	0.4214	0.6678	0.5517	0.5539	0.676
MCW330	0.6913	0.7043	0.7802	0.6926	0.7718	0.8573
MCW165	0.7055	0.6748	0.7821	0.7592	0.7405	0.7947
MCW103	0.8813	0.8324	0.8943	0.8550	0.8681	0.8940
MCW034	0.7863	0.7742	0.8513	0.7885	0.7398	0.8147
MCW081	0.6897	0.5153	0.6868	0.6108	0.6489	0.7335
MCW284	0.6650	0.6237	0.684	0.7069	0.7371	0.7730
MCW078	0.7744	0.7242	0.7235	0.7077	0.7666	0.8002
ADL268	0.7744	0.7242	0.7235	0.7077	0.7666	0.8002
ADL112	0.7681	0.7339	0.7793	0.7555	0.7547	0.8279

PIC: Polymorphic information content

### Heterozygosity values

The heterozygosity values are depicted in Table-3. The mean H_0_ over all loci was lowest for Bangalore Rural with a value of 0.855 and highest for Chickaballapura with a value of 0.868. The mean H_e_ over all loci was lowest for Bangalore rural with a value of 0.685 and highest for Chamrajnagara with a value of 0.803. The mean uH_e_ over all loci was lowest for Bangalore Rural with a value of 0.698 and highest for Chamrajnagara with a value of 0.815. The mean ‘F’ value over all loci was lowest for Bangalore Rural with a value of −0.256 and highest for Chamrajnagara with a value of −0.066.

**Table-3:** Summary of heterozygosity information over all loci for each population.

Ecotype	N	Na	N_e_	I	H_o_	H_e_	uHe	F
RNGR								
Mean	29.000	7.650	4.687	1.671	0.857	0.760	0.773	−0.121
SE	0.000	0.568	0.368	0.074	0.042	0.022	0.022	0.049
BNGRL								
Mean	28.000	5.600	3.640	1.381	0.855	0.685	0.698	−0.256
SE	0.000	0.336	0.286	0.081	0.041	0.031	0.031	0.046
CBR								
Mean	30.000	7.500	4.954	1.698	0.868	0.772	0.785	−0.117
SE	0.000	0.531	0.438	0.076	0.041	0.018	0.018	0.048
MYSORE								
Mean	30.000	6.600	4.100	1.532	0.857	0.731	0.743	−0.164
SE	0.000	0.328	0.290	0.066	0.041	0.021	0.022	0.048
MANDYA								
Mean	27.000	6.600	4.367	1.563	0.861	0.748	0.762	−0.137
SE	0.000	0.444	0.310	0.072	0.051	0.019	0.020	0.065
CHMNGR								
Mean	33.000	8.600	5.518	1.835	0.861	0.803	0.815	−0.066
SE	0.000	0.472	0.374	0.063	0.042	0.014	0.014	0.048

### Genetic distances

Among the 15 pairs of ecotypes studied, the chickens of Ramanagara and Chamrajnagara were most distant with a Nei’s genetic distance value of 0.22. The chickens of Bangalore rural and Mysore were least distant with a value of 0.056. The Ramanagara and Chamrajnagara pair had Nei’s genetic identity value of 0.802, which is least among all pairs of ecotypes. The Bangalore Rural and Mysore had maximum Nei’s genetic identity with a value of 0.946 among all the pairs of ecotypes (Table-4).

**Table-4:** Pairwise genetic identity (above the diagonal) and distance (below the diagonal) values.

Ecotype	RNGR	BNGRL	CBR	MYSORE	MANDYA	CHMNGR
RNGR	1.000 0.000	0.942	0.911	0.909	0.868	0.802
BNGRL	0.060	1.000 0.000	0.922	0.946	0.900	0.845
CBR	0.093	0.081	1.000 0.000	0.858	0.825	0.829
MYSORE	0.095	0.056	0.153	1.000 0.000	0.936	0.863
MANDYA	0.142	0.105	0.192	0.066	1.000 0.000	0.930
CHMNGR	0.220	0.169	0.187	0.148	0.073	1.000 0.000

Among the 15 pairs of ecotypes studied, the chicken of Ramanagara and Chamrajnagara were most distant with an unbiased Nei’s genetic distance value of 0.159. The chicken of Ramanagara and Bangalore Rural were least distant with a value of 0.011. The Ramanagara and Chamrajnagara pair had Unbiased Nei’s genetic identity value of 0.852 which is least among all pairs of ecotypes. The Ramanagara and Bangalore Rural had maximum unbiased Nei’s genetic identity with a value of 0.989 among all the pairs of ecotypes (Table-5). The genetic distance between populations provides a relative estimate of the time elapsed since the subdivisions existed as a single population and helps in characterizing the breeds or lines. Among the 15 pairs of ecotypes, Nei’s genetic distance was in the range of 0.056-0.22. The similar trend was observed for Nei’s unbiased genetic distances as well for the 15 pairs of ecotypes. The Nei’s standard genetic distances reported by Chang *et al*. [3] were moderate to high (0.59-0.93). The findings of Sangwon *et al*. [4] were in conformity with our genetic distance values. The genetic distance of such magnitude is predictable for the ecotypes which are not completely isolated from each other for a longer number of generations and they are also not subjected to differential selection pressures. The exchange of genes between populations homogenizes allele frequencies between populations and determines the relative effects of selection and genetic drift. High gene flow precludes local adaptation (i.e. the fixation of alleles, which are favored under local conditions), and will therefore also impede the process of speciation [8].

**Table-5:** Pairwise Nei’s unbiased genetic identity (above diagonal) and distance (below diagonal) values.

Ecotype	RNGR	BNGRL	CBR	MYSORE	MANDYA	CHMNGR
RNGR	1.000 0.000	0.989	0.966	0.958	0.919	0.853
BNGRL	0.011	1.000 0.000	0.969	0.988	0.945	0.890
CBR	0.035	0.031	1.000 0.000	0.905	0.875	0.882
MYSORE	0.043	0.012	0.100	1.000 0.000	0.986	0.912
MANDYA	0.084	0.056	0.134	0.014	1.000 0.000	0.988
CHMNGR	0.159	0.116	0.125	0.092	0.012	1.000 0.000

### Dendrogram

**Figure-2:**
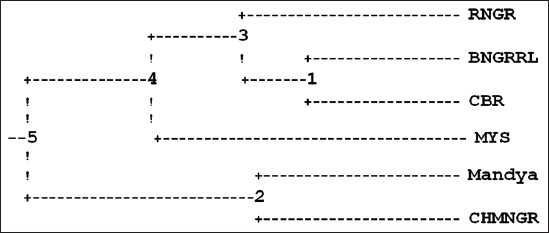
Dendrogram based Nei’s genetic distance for the six ecotypes of indigenous chicken.

The dendrogram was constructed on the basis of genetic distance [9] and neighbor-joining methods following un-weighted pair-group method using arithmetic averages. There were five main nodes from which the six ecotypes evolved on the basis 20 microsatellite markers used in this study (Figure-2). Nodes four and two drifted from five and were geographically isolated for a prolonged number of generations represented in time units of 2.24 and 4.03, respectively. The node three and Mysore drifted from four and were geographically separated for a prolonged number of generations represented in time units of 1.56 and 5.45, respectively. The Ramanagara ecotype and node one drifted from node three in the recent past and geographically isolated in time units of 3.88 and 1.10, respectively. Bangalore Rural and Chickaballapura ecotypes drifted from node one, evolved into current type by geographical isolation for a time unit of 2.79. Mandya and Chamrajnagara ecotypes were evolved from node two by drifting and geographical isolation for a time unit of 3.65 (Table-6). The accuracy of a reconstructed tree also depends on a tree making method and distance measures used. Nei [10] suggested that although this result is discouraging it must be accepted due to the stochastic nature of gene substitution and that one cannot be over confident about the evolutionary tree reconstructed from electrophoretic data. There were five main nodes from which the six ecotypes evolved on the basis of 20 microsatellite markers used in this study. Nodes four and two drifted from five and were geographically isolated for a prolonged number of generations represented in time units of 2.24 and 4.03 respectively. The node three and Mysore drifted from four, geographically separated for a prolonged number of generations represented in time units of 1.56 and 5.45 respectively. The Ramanagara ecotype and node one drifted from node three in the recent past and geographically isolated in time units of 3.88 and 1.10 respectively. Bangalore Rural and Chickaballapura ecotypes drifted from Node one, evolved into current type by geographical isolation for a time unit of 2.79. Mandya and Chamrajnagara ecotypes were evolved from node two by drifting and geographical isolation for time unit of 3.65. This study indicates that the four ecotypes Ramnagara, Bangalore Rural, Chickaballapura and Mysore are genetically identical due to their common ancestral evolution while, Mandya and Chamrajnagara ecotypes formed a relatively different cluster due to a separate common ancestral chicken population and less number of generations since drifting from bifurcation node.

**Table-6:** The branch lengths among five nodes and ecotypes of the dendrogram.

Nodes	Nodes/ecotypes	Branch length
5	4	2.24
4	3	1.56
3	Ramanagara	3.88
3	1	1.10
1	Bangalore rural	2.79
1	Chickaballapura	2.79
4	Mysore	5.45
5	2	4.03
2	Mandya	3.65
2	Chamrajnagara	3.65

### Wright’s F-statistics

The F_IS_ was in the range of −0.475 in MCW123 to 0.551 in MCW183. The F_IT_ was in the range of −0.430 in MCW123 to 0.598 in MCW183. The F_ST_ was in the range of 0.012 in MCW123 to 0.104 in MCW183. The F_ST_ values calculated by frequency option (Table-7) was highest (0.032) for Bangalore Rural and Chamrajnagara pair while lowest (0.011) for Mandya’s pairing with two other districts of Mysore division. Complimentary to the above trend, the number of migrants (N_m_) between the ecotype pairs was lowest (7.587) for Bangalore Rural and Chamrajnagara pair, while highest (around 22) for Mandya’s pairing with two other districts of Mysore division. Fixation indices give an idea about the population structure in terms of the inbreeding coefficient and population differentiation. The F and F_IS_ measures “the Ho expressed as a fraction of H_e_ and deviation from 1” for each of the locus/all loci in each ecotype and over all populations for each locus, respectively. The former takes into account individual locus H_0_ and H_e_ values while later considers mean H_0_ and H_e_ values of all ecotypes for each of the locus. They are influenced by the deviation of H_e_ from H_0_. The values were on the positive side for loci with lower H_0_ than H_e_ and are on the negative side for loci with higher H_0_ than H_e_. The values indicate the extent of fixation of alleles in each of the loci. The F values were positive for only three loci namely MCW183, MCW067 and MCW014 indicating relatively better allele fixation in these loci compared to remaining seventeen loci which had negative F values. The mean F per ecotype over all loci was lowest for Bangalore Rural with a value of −0.256 and highest for Chamrajnagara with a value of −0.066 indicating better allele fixation in Chamrajnagara ecotype among all ecotypes. The values were in conformity with the ranges reported by Pirany *et al*. [11]. The F_IS_ was in the range of −0.475 in MCW123 to 0.551 in MCW183. The range of FIS in the present study was wider compared to the reports of −0.29 to 0.38 and −0.135 to 0.137 by Pirany *et al*. [11], respectively. The difference can be ascribed to the difference in the microsatellite markers utilized, mutation rates in genetic groups and varied selection intensities for traits linked to microsatellite markers. The H_t_ is similar to H_e_ except that the former is a measure of expected heterozygosity over all the ecotypes while later is restricted to one population. The behavior of H_t_ values is similar to that of H_e_. The values are directly proportional to the polymorphism of loci. The similar range of H_t_ values was reported by Pirany *et al*. [11]. Fixation indices such as F_IS_, F_IT_ and F_ST_ give an idea about the population structure in terms of inbreeding coefficient and population differentiation [12].

**Table-7:** F-statistics and estimates of N_m_ over all ecotypes for each locus.

Locus	H_t_	Mean H_e_	Mean H_0_	F_IS_	F_IT_	F_ST_	N_m_
ADL020	0.745	0.728	0.737	−0.012	0.011	0.023	10.703
ADL023	0.777	0.752	0.771	−0.024	0.008	0.031	7.738
ADL176	0.682	0.661	0.937	−0.419	−0.375	0.031	7.849
MCW007	0.862	0.849	1.000	−0.178	−0.160	0.015	16.400
MCW041	0.785	0.764	0.972	−0.273	−0.238	0.028	8.782
MCW014	0.839	0.822	0.790	0.039	0.058	0.020	11.955
MCW183	0.516	0.462	0.207	0.551	0.598	0.104	2.159
ADL278	0.798	0.768	0.797	−0.038	0.001	0.037	6.446
MCW067	0.746	0.723	0.631	0.126	0.154	0.031	7.761
MCW104	0.805	0.773	0.932	−0.206	−0.157	0.040	5.955
MCW123	0.664	0.644	0.949	−0.475	−0.430	0.030	7.947
MCW330	0.816	0.781	0.972	−0.245	−0.192	0.043	5.590
MCW165	0.799	0.776	0.960	−0.238	−0.202	0.029	8.357
MCW103	0.893	0.882	1.000	−0.133	−0.120	0.012	21.264
MCW034	0.850	0.817	1.000	−0.224	−0.177	0.039	6.138
MCW081	0.713	0.691	0.728	−0.054	−0.021	0.032	7.681
MCW284	0.757	0.743	1.000	−0.346	−0.322	0.018	13.755
MCW078	0.811	0.780	0.906	−0.162	−0.118	0.038	6.328
ADL268	0.811	0.780	0.906	−0.162	−0.118	0.038	6.328
ADL112	0.814	0.798	1.000	−0.253	−0.229	0.019	12.807

The F_IT_ was in the range of −0.430 in MCW123 to 0.598 in MCW183 with a mean of −0.101 over all loci and ecotypes in the present study. For the interpretation of F_ST_, it has been suggested that a value lying in the range 0-0.05 indicates little genetic differentiation; a value between 0.05 and 0.15, moderate differentiation; a value between 0.15 and 0.25, great differentiation; and values above 0.25, very great genetic differentiation. Balloux *et al*. [12] elaborated that a F_ST_ of 0.05 will generally be considered as reasonably low, and investigators may interpret that structuring between sub populations is weak. While such an interpretation may turn out to be correct, it may also not be representative at all of the real population differentiation because of the fact that expectation of F_ST_, under complete differentiation will not always be one. In fact, in the great majority of cases, it will not be one because the effect of polymorphism (due to mutations) drastically deflates F_ST_ expectations. Hence, a seemingly low F_ST_ of 0.05 may, in fact, indicate very important genetic differentiation. This point was already stressed by Barton and Hewitt [8], who wrote that differentiation is by no means negligible if F_ST_ is as small as 0.05 or even less. The F_ST_ was in the range of 0.012 in MCW123 to 0.104 in MCW183 with a mean of 0.033 over all loci and ecotypes. The mean FIS, FIT and F_ST_ for all the microsatellites together in the overall population was −0.18, −0.085 and 0.083, respectively for five chicken populations including two indigenous breeds and three pure lines of Indian Rural synthetics in the reports of Chatterjee *et al*. [13]. The F_ST_ values in the present study were lower than those reported by FAO and Nei [9,14].

## Conclusions

Twenty microsatellite markers based genetic diversity study on six indigenous ecotypes indicated lower genetic distances as well as lower F_ST_ values compared to those values between distinguished breeds reported. There were two main clusters, which differentiated into six ecotypes. They may differentiate into more distinct varieties if bred in isolation for a longer number of generations.

## Authors’ Contributions

This is part of the Ph.D. thesis submitted by the first author, consequently research, analysis and manuscript preparation was done by the same. HNNM suggested material collection plan, MRJ instructed format for recording of laboratory data, CSN suggested PCR trouble shooting methods, AMK helped in caliberating Thermal cycler for all primer pairs and SMB provided the key information for interpretation of laboratory results. All authors read and approved the final manuscript.
